# Cardiovascular Reactivity to a Novel Stressor: Differences on Susceptible and Resilient Rats to Social Defeat Stress

**DOI:** 10.3389/fphys.2021.781447

**Published:** 2022-02-16

**Authors:** Gessynger Morais-Silva, Lucas Gomes-de-Souza, Willian Costa-Ferreira, Jacqueline C. Pavan, Carlos C. Crestani, Marcelo T. Marin

**Affiliations:** ^1^Laboratory of Pharmacology, School of Pharmaceutical Sciences of Araraquara, São Paulo State University, Araraquara, Brazil; ^2^Joint Graduate Program in Physiological Sciences (PIPGCF), UFSCar/UNESP, Araraquara, Brazil

**Keywords:** social defeat stress, depression, restraint stress, heart rate, blood pressure, resilience, susceptibility

## Abstract

Prolonged and heightened responses to stress are known factors that influence the development of mood disorders and cardiovascular diseases. Moreover, the coping strategies related to the experience of adverse events, i.e., resilience or the susceptibility to stress, are determinants for the individual risk of developing such diseases. Susceptible rats to the social defeat stress (SDS), identified by the social interaction test (SIT), show behavioral and cardiovascular alterations after SDS exposure that are not found in resilient rats. However, it is not elucidated yet how the cardiovascular system of susceptible and resilient phenotypes responds to a new stressor after SDS exposure. Thus, using the SDS exposure followed by the SIT, we evaluated heart rate, blood pressure (BP), tail skin temperature, and circulating corticosterone responses to an acute session of restraint stress in susceptible and resilient rats to SDS. Susceptible rats showed resting tachycardia and exaggerated BP response to restraint stress, while resilient rats did not present such alterations. In contrast, both phenotypes showed increased plasma corticosterone and a drop in tail skin temperature to restraint stress, which was similar to that observed in control animals. Our results revealed an increased cardiovascular reactivity in response to a new stressful stimulus in susceptible rats, which might be related to a greater risk for the development of cardiovascular diseases.

## Introduction

Immediate responses of the cardiovascular system to acute stress are well-recognized changes that prepare the challenged organism to cope with a threat. They are comprised of a set of autonomic nervous system changes that lead to increased heart rate (HR) and blood pressure (BP) values concurrently (Crestani, 2016) and a shift in the baroreflex set point (Crestani et al., 2010). In addition to such changes, the sympathetically mediated cutaneous vasoconstriction causes a drop in tail skin temperature (Busnardo et al., 2019). In contrast, prolonged and heightened cardiovascular responses to stress are considered a risk factor and could lead to diseases (Hughes et al., 2018).

There is a strong correlation between cardiovascular diseases and depression. Depression is an independent risk factor for the development and aggravation of a wide range of cardiovascular diseases (Suls, 2018). In this sense, stress experience is an important risk factor for the development of both cardiovascular diseases and psychiatric disorders (Penninx, 2016).

In animal models to study stress, chronic exposure to adverse stimuli increases depressive-like behaviors in a wide range of behavioral tests and induces cardiovascular dysfunctions, such as resting tachycardia, increased BP, and the emergence of arrhythmias (Deussing, 2006; Sgoifo et al., 2014; Crestani, 2016). The social defeat stress (SDS) model is an ethologically relevant protocol to study stress in rodents, with a strong resemblance to psychosocial stress that is experienced by human beings. Chronic exposure to SDS leads to increased anxiety-like behaviors when tested in the novelty suppression of feeding test or the elevated plus-maze apparatus; depressive-like behaviors in the forced swim test, sucrose consumption and tail suspension tests; social avoidance; and autonomic and electrophysiological changes in the heart (Berton et al., 2006; Venzala et al., 2012; Sévoz-Couche et al., 2013).

Individual traits are an important determinant of stress exposure as a risk factor, since not every individual experiencing stress gets sick (Cohen et al., 2007). Such traits seem to be related to the ability of each organism to cope with harmful experiences, recognized as its resilience or susceptibility to stress. Recently, chronic SDS (CSDS) exposure along with the social interaction test (SIT) started to be used, initially in mice, to identify and study susceptible and resilient phenotypes to stress. In this paradigm, susceptible mice show decreased social interaction and increased depressive-like behaviors, while resilient mice do not show such alterations (Krishnan et al., 2007). In our lab, we developed a modified protocol of SDS exposure followed by the SIT, which allowed the study of different coping phenotypes to SDS in rats. Susceptible rats identified by this protocol showed social avoidance to an unknown and non-aggressive conspecific, increased coat state deterioration during SDS exposure, and an increase in immobility during the forced swim test (Morais-Silva et al., 2019). These behavioral changes were followed by cardiovascular alterations, such as increased sympathetic activity and impaired baroreflex effectiveness during rest that, in turn, lead to decreased HR variability and resting tachycardia (Morais-Silva et al., 2019). Despite these pieces of evidence, it is not elucidated yet how the cardiovascular system of susceptible and resilient phenotypes responds to a new stressor after CSDS. Thus, this study aimed to evaluate the cardiovascular and plasma corticosterone responses to a new stressor in susceptible and resilient rats to SDS.

## Materials and Methods

### Experimental Subjects

A total of 22 male Wistar rats (230–250 g) obtained from the Sao Paulo State University (UNESP) animal breeding facility (Botucatu, Brazil) were used as experimental subjects. After arriving, animals were habituated to our animal facility for 7 days prior to being used in the experiments. Animals were maintained in a controlled temperature room (23 ± 2°C), on a reverse 12/12 h light–dark cycle (lights on at 10 p.m.), in groups of four or five animals per cage (32 cm × 40 cm × 16 cm), with water and food *ad libitum*. Stress and control rats were housed in separated cages (i.e., animals were allocated in the “control” and “stressed” groups before the beginning of the experiments). Behavioral experiments were conducted during the dark phase of the cycle (randomly between 1 p.m. and 4 p.m.), since aggressors were more active and consequently more aggressive during this period, whereas cardiovascular measurements were conducted during the light phase of the cycle (11 p.m.–4 a.m.) to reduce the impact of the locomotion and exploratory behavior of the animals to the recording of the cardiovascular parameters (Morais-Silva et al., 2019).

A total of five male Long Evans rats (>400 g) were used as aggressors in the SDS. Aggressors were kept in an adjacent room, in a permanent colony with a sexually mature and viable female Long Evans rat (32 cm × 40 cm × 16 cm). The room was maintained at a controlled temperature (23 ± 2°C) and in a reverse 12/12 h light–dark cycle. Water and food were offered *ad libitum*. Before 1 h to each agonistic encounter, the female and pups were removed from the home cage of the aggressor and kept undisturbed in an adjacent room, while the aggressors were moved to the experimental room.

All procedures involving animals were conducted according to the principles of the National Council for Animal Experiments Control (CONCEA), based on the National Institutes of Health Guidelines for the Care and Use of Laboratory Animals and approved by the local ethics committee for animal care and use (CEUA FCF/CAr) under the protocol number 01/2017.

### Social Defeat Stress

The SDS consisted of exposing the experimental animals (named intruders) to the home-cage of an aggressive male Long Evans rat (named residents). This was based on the protocol previously described by our lab (Morais-Silva et al., 2019).

Intruders were exposed to four agonistic encounters, every other day, for 7 days. Each agonistic encounter was comprised of three phases as follows: first, in the incitation phase, intruders were exposed to the cage of the resident protected by a metal-grid cage for 5 min to stimulate the aggressive behavior of the resident. Next, intruders were taken out of the protective cage and placed in direct contact with the resident rat for 10 min (defeat phase). Finally, intruders were put back in the metal-grid cage for 10 min (post-defeat phase). All the intruders from the same cage were exposed to SDS at the same time. At the end of the post-defeat phase, the intruders were returned to their home-cages for 48 h, when the next defeat occurred. Intruders were exposed to a different resident (Long Evans rat) in each defeat episode.

Stressed and control animals were manipulated for physical state evaluation and weighing right before each defeat. Control animals were kept undisturbed (except for physical evaluation and cage cleaning) in the animal facility during the SDS sessions.

### Physical State Evaluation

Coat state deterioration was evaluated as previously described (Costa-Ferreira et al., 2019; Morais-Silva et al., 2019). Accordingly, right before the first defeat episode and the SIT, the fur state of control and stressed animals was evaluated and was scored using a scale ranging from 3 (well-cared and healthy fur) to 0 (sick and dirty fur, showing signs of intense hair loss and piloerection). The coat state deterioration score was expressed as the difference between coat state scores before the SIT and first defeat episode.

### Social Interaction Test

The SIT was performed as previously described (Morais-Silva et al., 2019). Accordingly, control and stress rats were tested as follows: In the first phase (no target), animals were allowed to freely explore the arena for 2.5 min with the target enclosure empty. Then, experimental rats were removed from the arena for 30 s, and the target enclosure was changed to one containing a Long Evans male rat (novel and non-aggressive). The animals were then placed again in the arena for 2.5 min. Time spent in the interaction and avoidance zones and latency to the first entry in the interaction zone during no target and target phases were evaluated using the behavioral analysis software ANY-Maze (Stoelting Co., Wood Dale, IL, United States).

The phenotype of the intruders was identified based on the *k*-means cluster analysis of the interaction ratio (IR), using two subgroups classification (resilient and susceptible), as previously described (Morais-Silva et al., 2019).

### Femoral Artery Cannulation

Right after the SIT (2 h), animals were anesthetized with tribromoethanol (250 mg/kg) intraperitoneally (1 ml/kg) and one catheter [4 cm segment of polyethylene (PE)-10 heat bound to a 13 cm segment of PE-50; Clay Adams, Parsippany, NJ, United States] was implanted inside the femoral artery for blood sampling and recording of the pulsatile arterial pressure (PAP). The catheter was tunneled under the dorsum skin, exteriorized near the neck, and filled with a solution of heparin (50 UI/ml, Hepamax-S, Blausiegel, Cotia, Brazil) diluted in saline (0.9% NaCl). After the surgery, animals were maintained in the recording room, in individual cages, for the rest of the experiment (Morais-Silva et al., 2019). A prophylactic dose of a non-steroidal anti-inflammatory [flunixin meglumine, 2.5 mg/kg, 0.1 ml/100 g, subcutaneous (s.c.), Chemitec Agro-Veterinária Ltd., São Paulo, Brazil] and antibiotics [a formulation containing streptomycin and penicillin, 560 mg/kg, 0.2 ml, intramuscular (i.m.), Zoetis Ltd., Campinas, Brazil] were administered to the animals for pain control and to prevent infections.

### Recording of the Cardiovascular Parameters

After the surgery (48 h), the catheter implanted into the femoral artery was connected to a pressure transducer (DPT100, Utah Medical Products Inc., Midvale, UT, United States). PAP was recorded for 10 min before restraint stress and for 30 min during stress (detailed described below) using an amplifier connected to a digital acquisition board (ADInstruments, Dunedin-OTA, NZ) and LabChart PRO software (ADInstruments, Dunedin). The mean arterial pressure (MAP) and HR were derived from the PAP (Morais-Silva et al., 2019). The mean of the MAP and HR values each 2 min was calculated for time-course analysis.

### Acute Restraint Stress

Cardiovascular alterations in response to a novel stress exposure were evaluated using the acute restraint stress paradigm based on the protocol already described but with modifications (Gomes-de-Souza et al., 2019). Initially, animals were connected to the pressure transducer and kept undisturbed for 30 min. The last 10 min were used for the evaluation of the basal values of MAP and HR. After that, they were introduced into cylindrical plastic tubes (6.5 cm diameter × 15 cm length; ventilated by 1 cm holes covering 20% of the tube surface) for 30 min. MAP and HR were expressed as changes from the mean basal value recorded 10 min before restraint onset. A mean of overall restraint effect (the mean of all time points obtained during restraint stress exposure) was analyzed to facilitate visualizing group differences in restraint stress response.

### Cutaneous Tail Temperature

Ten minutes before, right after, 15 and 30 minutes after introducing animals into the restraint tube, thermal images were recorded to analyze stress-induced changes in cutaneous tail temperature. The cutaneous tail temperature was evaluated using a thermal camera (IRI4010, InfraRed Integrated Systems Ltd., Northampton, United Kingdom). Cutaneous temperature for each time point was calculated as the mean of five point values on the tail of the animal obtained from the whole tail. The room was maintained at a controlled temperature (23 ± 1°C) during the whole experiment.

### Plasma Corticosterone Quantification

In the first hour of the light phase of the cycle, a blood sample (100 μl) was collected from the femoral artery for the determination of basal plasma corticosterone. After 30 min of the beginning of the restraint stress, another blood sample (100 μl) was collected for the evaluation of restraint stress effects. After each sampling, the catheter was filled again with heparin solution (50 UI/ml), which was discarded before blood sampling. Blood samples were collected in plastic tubes containing heparin solution (5 μl, 5,000 UI/ml) and were centrifuged (2,000 × *g* for 10 min, 4°C). The plasma was collected and stored frozen (−80°C) until quantification. Plasma corticosterone concentration was estimated using a commercial corticosterone enzyme-linked immunosorbent assay (ELISA) following the instructions of the manufacturer (Cayman Chemical, Ann Harbor, MI, United States). Plasma samples were diluted in ELISA buffer (1:250) before quantification.

### Statistical Analysis

Data were expressed as mean ± SE and analyzed by one-way or repeated-measures analysis of variance (ANOVA). In cases where ANOVA showed significant differences (*p* ≤ 0.05), the Newman–Keuls *post hoc* test was performed. For identification of the stress-coping phenotypes, IR values of the stressed animals were classified into two clusters, namely, maximizing between-cluster distance and minimizing within-cluster distance, using the *k*-means cluster analyses. Statistics were performed using Statistica 7.1 software (StatSoft, Inc., Tulsa, OK, United States), and graphs were constructed in the GraphPad Prism 7 software (GraphPad Software Inc., La Jolla, CA, United States).

## Results

### Susceptible Rats Show Social Avoidance and Increased Depressive-Like Behavior After Social Defeat

**FIGURE 1:**
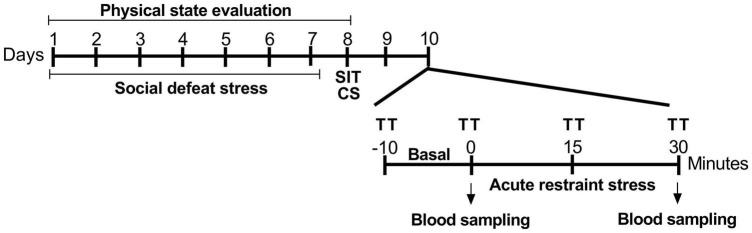
Experimental procedure timeline. SDS was performed on alternative days for 7 days, for a total of four aggressive encounters. After 24 h, stress coping phenotypes were identified using the social interaction test. After the phenotyping, animals were cannulated to the recording of cardiovascular parameters during restraint stress. SDS, social defeat stress; SIT, social interaction test; CS, cannulation surgery; TT, cutaneous tail temperature.

**FIGURE 2:**
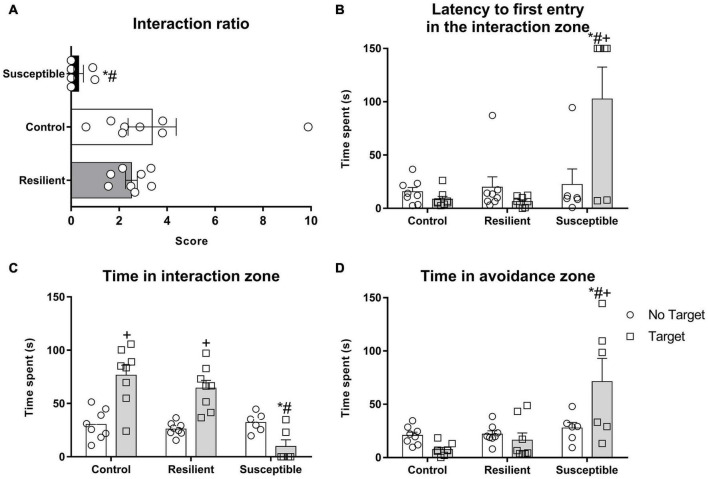
Social parameters of the susceptible and resilient phenotypes in the SIT. The test was performed 24 h after the last SDS. Bars represent means + SEM (*n* = 6–8 animals per group). **(A)** Interaction ratio (IR). **(B)** Latency to the first entry in the interaction zone. **(C)** Time spent in the interaction zone. **(D)** Time spent in the avoidance zone. *p < 0.05 relative to control group; ^#^*p* < 0.05 relative to resilient group; ^+^*p* < 0.05 relative to time spent in the no target phase of the same group.

The experimental timeline is depicted in Figure 1. As expected, susceptible and resilient rats showed large differences regarding social interaction. Socially defeated susceptible rats showed decreased IR compared with the resilient and control groups (Figure 2A). One-way ANOVA of the IR revealed a significant effect for phenotype factor (*F*_2,19_ = 5.18, *p* < 0.05). The Newman–Keuls *post hoc* test revealed a decrease in IR of susceptible groups compared with the control (*p* < 0.05) and resilient groups (*p* < 0.05).

In the SIT, susceptible rats showed increased latency to enter the interaction zone for the first time when the target is present (Figure 2B). Repeated measures ANOVA showed a significant effect for phenotype factor (*F*_2,19_ = 17.48, *p* < 0.001) and for the interaction between phenotype and session (*F*_2,19_ = 5.97, *p* < 0.01). The Newman–Keuls *post hoc* test revealed an increased latency for susceptible animals to enter the interaction zone for the first time when the target is present when compared with the no target session (*p* < 0.01) and to the control (*p* < 0.001) and resilient groups (*p* < 0.0001) during the target session.

Time spent in the interaction zone (Figure 2C) was also different between phenotypes. Repeated measures ANOVA showed a significant effect for phenotype factor (*F*_2,19_ = 12.61, *p* < 0.001), for session (*F*_1,19_ = 16.17, *p* < 0.001), and for their interaction (*F*_2,19_ = 12.28, *p* < 0.001). The *Post hoc* test revealed a decrease in time spent in the interaction zone when target is present in the susceptible rats compared with the control (*p* < 0.001) and resilient rats (*p* < 0.001).

Rats that were susceptible to the SDS spent more time in the avoidance zone of the SIT apparatus (Figure 2D) when the target is present. Repeated measures ANOVA showed a significant effect for phenotype factor (*F*_2,19_ = 11.19, *p* < 0.001) and for the interaction between phenotype and session (*F*_2,19_ = 5.59, *p* < 0.05). The *Post hoc* test revealed a significant amount of time spent in the avoidance zone when the target is present by the susceptible group when compared with the no target session (*p* < 0.001) and with the control (*p* < 0.001) and resilient rats (*p* < 0.001).

There was a decrease in body weight gain (Table 1) in socially defeated rats regardless of the coping phenotype relative to controls. One-way ANOVA showed a significant effect of phenotype (*F*_2,19_ = 6.34, *p* < 0.01). The *Post hoc* test revealed that resilient (*p* < 0.01) and susceptible (*p* < 0.05) rats gained less weight compared with controls. There were no significant differences in initial or final absolute body weight among groups (Table 1). Susceptible rats showed an increase in coat state deterioration after SDS exposure (Table 1). One-way ANOVA showed a significant effect for phenotype factor (*F*_2,19_ = 6.30, *p* < 0.01), and Newman–Keuls revealed a significant difference when comparing the susceptible rats with the control (*p* < 0.05) and resilient rats (*p* < 0.01).

**TABLE 1:** Body weight, body weight gain, and coat state deterioration of resilient and susceptible phenotypes to social defeat stress (SDS).

		Control	Resilient	Susceptible
Body weight (g)	Initial	232 ± 7	229 ± 3	233 ± 10
	Final	278 ± 10	254 ± 7	262 ± 15
Body weight gain (g)		46 ± 3	25 ± 5*	30 ± 6*
Coat state deterioration		−0.25 ± 0.13	−0.31 ± 0.13	−0.92 ± 0.15*^#^

*Numbers represent means ± SEM (n = 6–8 animals per group).*

*p < 0.05 relative to control group.

*^#^p < 0.05 relative to resilient group.*

### Susceptible Rats Show Resting Tachycardia and Exaggerated Mean Arterial Pressure Response to Restraint Stress

Resting HR was different across coping phenotypes (Table 2). One-way ANOVA showed a significant effect for phenotype factor (*F*_2,19_ = 5.95, *p* < 0.01). The Newman–Keuls *post hoc* test revealed increased HR values in susceptible animals compared with the control (*p* < 0.05) and resilient rats (*p* < 0.01). There were no significant alterations in resting MAP (Table 2).

**TABLE 2:** Resting heart rate, mean arterial pressure, and tail skin temperature of resilient and susceptible phenotypes to SDS.

	Control	Resilient	Susceptible
Heart rate (bpm)	346 ± 11	338 ± 10	387 ± 10*^#^
Mean arterial pressure (mmHg)	108 ± 4	109 ± 2	101 ± 5
Tail skin temperature (°C)	27.6 ± 0.6	28.0 ± 0.3	27.6 ± 0.3

*Numbers represent means ± SEM (n = 6–8 animals per group).*

*p < 0.05 relative to control group.

*^#^p < 0.05 relative to resilient group.*

**FIGURE 3:**
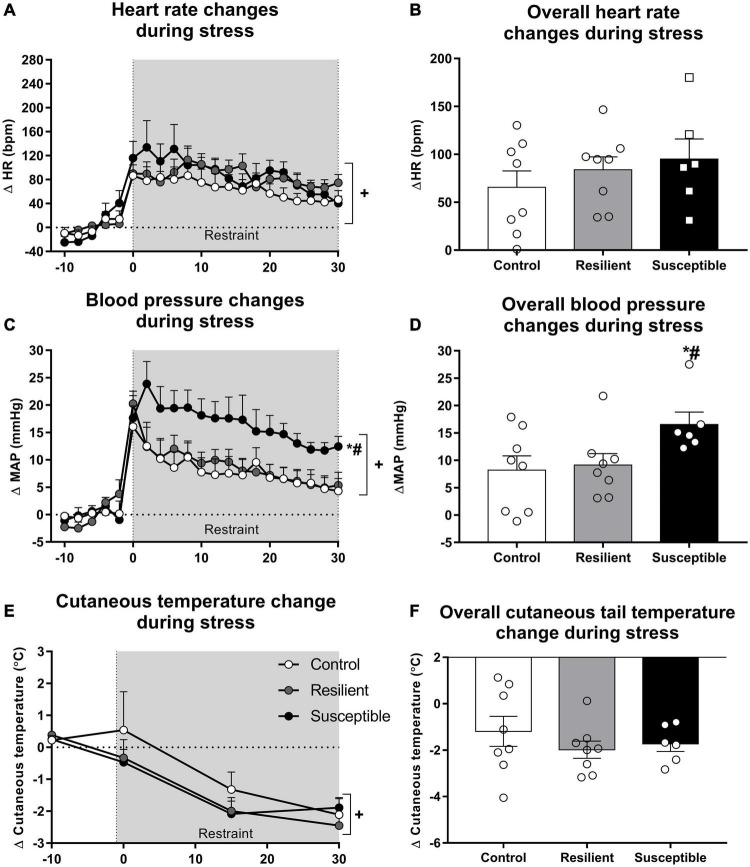
Heart rate (HR), blood pressure, and cutaneous tail temperature changes during restraint stress in resilient and susceptible phenotypes to SDS. Animals were cannulated 48 h after the last social defeat episode, and cardiovascular parameters were recorded 24 h later. Points represent means + SEM (*n* = 6–8 animals per group). The values were expressed as changes from the mean basal value, obtained for 10 min. **(A)** HR changes during restraint stress exposure. **(B)** Overall HR changes during restraint stress exposure. **(C)** Mean arterial pressure (MAP) changes during restraint stress exposure. **(D)** Overall MAP changes during restraint stress exposure. **(E)** Cutaneous tail temperature changes during restraint stress exposure. **(F)** Overall cutaneous tail temperature changes during restraint stress exposure. *p < 0.05 relative to control group; ^#^*p* < 0.05 relative to resilient group; ^+^significant restraint stress effect.

Tachycardic response to a novel stress exposure was not different among groups (Figures 3A,B). Repeated measures ANOVA showed a significant effect only for the restraint factor (*F*_20,380_ = 22.52, *p* < 0.001), indicating a significant increase in HR during restraint stress exposure (Figure 3A). Analysis of the overall restraint stress effect also did not show any significant difference in the HR response to restraint stress between the phenotypes (Figure 3B).

Susceptible rats had increased MAP response to restraint stress when compared with the control and resilient rats (Figures 3C,D). Repeated measures ANOVA showed a significant effect for the restraint factor (*F*_20,380_ = 37.18, *p* < 0.001) and for the interaction between phenotype and restraint (*F*_40,380_ = 1.85, *p* < 0.01) (Figure 3C). The Newman–Keuls *post hoc* test revealed significant differences in the increase in MAP between the susceptible and control groups and the susceptible and resilient groups in the time points 2, 4, 6, 8, 10, 12, 14, and 16 min after the beginning of the restraint stress exposure (*p* < 0.05). Moreover, only susceptible animals showed increased MAP compared with their baseline values at the end of the restraint stress exposure (30 min timepoint; *p* < 0.05). One-way ANOVA of the overall restraint stress effect on MAP showed a significant effect for phenotype (*F*_2,19_ = 3.32, *p* = 0.05). Susceptible rats showed increased pressor response to restraint stress when compared with the control (*p* = 0.05) and resilient rats (*p* < 0.05) (Figure 3D).

Tail cutaneous temperature changes in response to stress were not altered among the phenotypes (Figures 3E,F). One-way ANOVA did not show significant differences for the absolute tail skin temperature at rest (Table 2). Repeated measures ANOVA showed a significant effect for the restraint factor only (*F*_4,76_ = 20.27, *p* < 0.001), showing a significant decrease in tail temperature during restraint stress (Figure 3E). Analysis of the overall restraint stress effect on skin temperature did not show any significant differences (Figure 3F).

### Coping Phenotypes to Social Defeat Do Not Show Differences in Stress-Induced Plasma Corticosterone Increase

**FIGURE 4:**
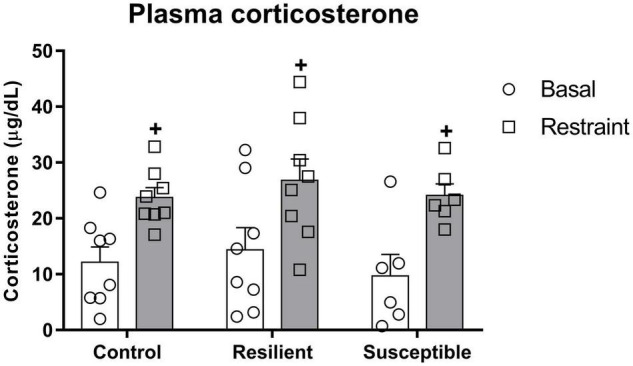
Plasma corticosterone levels in susceptible and resilient rats to SDS in response to restraint stress exposure. Blood samples were collected 10 min before and right after 30 min of restraint stress. Bars represent means + SEM (*n* = 6–8 animals per group). ^+^*p* significant restraint stress effect.

There was a significant effect for the restraint factor (*F*_1,19_ = 40.94, *p* < 0.001) on plasma corticosterone (Figure 4), indicating an increase in plasma corticosterone evoked by restraint stress. In contrast, it was not different among phenotypes.

## Discussion

Susceptible individuals to SDS, which shows increased depressive-like behavior in the SIT and coat state evaluation, have dysregulated resting HR and pressor response to stress, as shown by resting tachycardia and an exaggerated MAP response to restraint stress exposure, which might be mediated by hemodynamic changes in cutaneous beds.

The coat state deterioration index could be used as a simple method to evaluate depressive-like behavior in rodents since it reflects important alterations in grooming patterns, which are related to chronic stress experience (Alonso et al., 2004; Kalueff and Tuohimaa, 2005; Smolinsky et al., 2009). Chronic stress exposure increases coat state deterioration, which is reversed only by chronic treatment with antidepressants (Ibarguen-Vargas et al., 2008; Law et al., 2016; Mendez-David et al., 2017). Moreover, mice strains susceptible to stress-induced behavioral alterations present high levels of coat deterioration (Pothion et al., 2004; Yalcin et al., 2008; Nasca et al., 2014), while treatment-resistant mice do not show a reversal of deterioration scores after being treated with antidepressants (Isingrini et al., 2010). A recent study from our lab also showed that susceptible rats to SDS presented increased coat state deterioration (Morais-Silva et al., 2019).

Similar to a previous report from our lab (Morais-Silva et al., 2019), susceptible rats were tachycardic when cardiovascular parameters were obtained during the rest. Others have found similar alterations in resting HR in chronically stressed rodents (Carnevali et al., 2012; Sévoz-Couche et al., 2013; Crestani, 2016). Elevated values of HR increase the risk of developing cardiovascular diseases and are related to the difficulty to recover from such diseases (Boudoulas et al., 2015; Lindgren et al., 2018; Tadic et al., 2018). Accordingly, it was reported that mice exposed to chronic psychologic stress have increased resting HR and decreased endothelial function, which leads to bigger ischemic damage when the middle cerebral artery is occluded (Custodis et al., 2011). In rabbits, in which atherosclerotic plaques were surgically induced (van Hoof et al., 2017), monkeys (Beere et al., 1984; Kaplan et al., 1987), and apolipoprotein E-deficient mice (Custodis et al., 2008) fed with an atherogenic diet, lowering HR was beneficial for the cardiovascular system and retarded atherosclerotic plaque formation.

Exaggerated pressure response to acute stress challenge has been described in the literature in the animal models of depression (Grippo et al., 2002, 2010; Nalivaiko, 2011; Cudnoch-Jedrzejewska et al., 2014; McNeal et al., 2014; Benini et al., 2020) but not consistently. In men, social discrimination was related to increased cardiovascular reactivity during a stress session (Richman et al., 2007), while a positive emotional style accelerated the cardiovascular recovery after an acute stressor (Bostock et al., 2011). Our results showed an exaggerated MAP response exclusively in the susceptible phenotype. It seems to indicate an impairment in the cardiovascular response of animals to stress, which could be harmful to these animals when facing a new aversive situation. Our findings are supported by previous evidence that early life stress, which is a risk factor for developing cardiovascular diseases and psychiatric disorders during adulthood (Murphy et al., 2017), increased cardiovascular reactivity to acute restraint stress in borderline hypertensive Wistar-Kyoto (Sanders and Anticevic, 2007) and Sprague-Dawley rats (Igosheva et al., 2004) during adulthood. Specifically, we showed a prolonged increase in the MAP of the susceptible phenotype in response to a new stressor exposure, which could contribute, when repeated, to the development of cardiovascular diseases due to vascular remodeling (Schwartz et al., 2003; Treiber et al., 2003; Gasperin et al., 2009). In this sense, systolic BP (SBP) reactivity to stress, as calculated by the mean change in SBP during stressful tasks, is correlated to carotid intima-media thickness, a marker of cardiovascular disorder risk, in adolescents and adults (Jennings et al., 2004; Roemmich et al., 2011; Lambiase et al., 2012; DuPont et al., 2021).

Based on our results, we cannot say whether the exaggerated response to restraint stress is related to SDS exposure in susceptible animals or if this response to the restraint stress exposure is an intrinsic characteristic of the susceptible rats to the SDS. In this regard, acute stress elicits varying hemodynamic alterations in both humans and rats. Some individuals, designated as vascular responders, show a decrease in cardiac output together with increased systemic vascular resistance when exposed to acute stress, while others, designated as mixed responders, show an increase in both cardiac output and systemic vascular resistance (Turner et al., 1992; Knuepfer et al., 1993, 2001). Interestingly, rats designated as vascular responders are predisposed to a sustained stress-induced increase in BP when exposed to chronic stress (Muller et al., 2001), while depressive symptoms are associated with increased systemic vascular resistance and decreased cardiac output in response to a motor and mental task (Matthews et al., 2005; Salomon et al., 2009). While such results are interesting and seem to be related to cardiovascular responses to stress, how such hemodynamic alterations are related to our results remains unknown.

The exaggerated pressure response to a new stressor in susceptible rats could be related to increased sympathetic activity. Accordingly, the restraint stress-evoked tachycardia and hypertension are mediated by sympathetic activation (dos Reis et al., 2014). We reported previously that susceptible rats to SDS presented increased sympathetic tone to the heart at rest (Morais-Silva et al., 2019). Therefore, the exaggerated MAP response to restraint in susceptible rats to SDS seems to be related to facilitation in the sympathetic activity. In this sense, the difference in restraint-evoked MAP increase indicates an enhanced total peripheral resistance (TPR) during restraint stress in susceptible animals. This idea is supported by the absence of changes in tachycardiac response. Thus, although we have not assessed the cardiac output, the similar HR response indicates that the change in pressor effect is mainly related to vascular resistance. Nevertheless, we did not find differences in tail skin temperature drop between the experimental groups. However, the absence of change in skin temperature does not necessarily exclude an involvement of hemodynamic changes in cutaneous bed in the exaggerated pressor response to restraint stress once enhanced sympathetically mediated cutaneous vasoconstriction might have been offset by the exaggerated increase in MAP, so that the drop in tail skin temperature was similar in susceptible animals. Furthermore, we cannot exclude an involvement of changes in other beds once the hemodynamic changes during stressful events, such as restraint stress, include vasoconstriction of the splanchnic, renal, cutaneous, and celiac vascular beds, which are followed by vasodilatation of the skeletal muscle vasculature (Iriuchijima et al., 1982; Kirby et al., 1987; Kapusta et al., 1989; Zhang et al., 1992, 1996; Anderson and Overton, 1994; Schadt and Hasser, 1998; Blessing, 2003; Mohammed et al., 2014). Therefore, a possible enhanced TPR during restraint stress in susceptible animals might be related to an impairment in skeletal muscle vasculature vasodilation and/or enhanced vasoconstriction in cutaneous and other beds. In this sense, it is worth mentioning that the vascular segments seem to be differently affected by emotional stress (Kume et al., 2020), so that the different beds might be differently impacted by SDS. Therefore, further studies assessing the hemodynamic changes in different beds are necessary to elucidate the mechanisms related to the enhanced pressor response in susceptible animals. The concomitant increases in HR and BP during stress are elicited to prepare organisms facing harmful situations and are also finely controlled by the autonomic nervous system to not exceed a physiological range. Dysregulations of this control could lead to adverse cardiovascular events, including cardiac arrhythmias (Paton et al., 2005).

Hypothalamic-pituitary-adrenal (HPA) axis of both susceptible and resilient rats seems to be similarly activated during a novel stress exposure since we did not find significant differences in the phenotype regarding the basal plasma corticosterone and its increase in response to restraint stress exposure. The lack of difference between the phenotypes in basal plasma corticosterone difference is contrary to our previous study where we found an increase in basal plasma corticosterone in resilient rats (Morais-Silva et al., 2019). These findings are intriguing since several studies have reported altered HPA axis activity in depressed patients (Holsboer, 2000) and chronic administration of corticosterone could be used as an animal model of depression since it increases depressive-like behavior in rodents (Zhao et al., 2008). Nevertheless, the present findings are in line with previous evidence that rats identified as susceptible or resilient to SDS based on the latency to assume a submissive posture during a defeat episode did not show differences in corticosterone release during restraint stress exposure (Wood et al., 2010). Regarding our previous study (Morais-Silva et al., 2019), differences in the experimental timeline could explain the discrepancy between the two studies. In this study, we collected the plasma for corticosterone measurement 48 h after the cannulation surgery and compared it with 24 h in the previous study. Thus, in the first study, the changes could be related to the interaction between the phenotype and surgery procedure, different from this study. Therefore, more studies should be conducted to address the importance of HPA axis changes in the etiology of depression evoked by SDS.

In summary, the susceptible rats to SDS have exaggerated MAP response to restraint stress exposure. The susceptible phenotype shows increased depressive-like together with disrupted cardiovascular parameters, reinforcing the relationship between cardiovascular diseases and depression. The use of the social defeat paradigm and SIT to identify coping phenotypes seems to be a valid tool to study the etiology and new treatment strategies for psychiatric and cardiovascular diseases related to stress.

## Data Availability Statement

The raw data supporting the conclusions of this article will be made available by the authors, without undue reservation.

## Ethics Statement

The animal study was reviewed and approved by all procedures involving animals were conducted according to the principles of the National Council for Animal Experiments Control (CONCEA), based on the National Institutes of Health Guidelines for the Care and Use of Laboratory Animals and approved by the local ethics committee for animal care and use (CEUA FCF/CAr) under the protocol number 01/2017.

## Author Contributions

GM-S and MM conceived and designed the experiments. GM-S, LG-D-S, WC-F, and JP performed the experiments and analyzed the data. GM-S, LG-D-S, WC-F, CC, and MM interpreted the results. GM-S prepared the draft. GM-S, CC, and MM edited and revised the manuscript. All authors approved the final version of the manuscript.

## Conflict of Interest

The authors declare that the research was conducted in the absence of any commercial or financial relationships that could be construed as a potential conflict of interest.

## Publisher’s Note

All claims expressed in this article are solely those of the authors and do not necessarily represent those of their affiliated organizations, or those of the publisher, the editors and the reviewers. Any product that may be evaluated in this article, or claim that may be made by its manufacturer, is not guaranteed or endorsed by the publisher.
